# Impact of positive end expiratory pressure on cerebral hemodynamic in paediatric patients with post-traumatic brain swelling treated by surgical decompression

**DOI:** 10.1371/journal.pone.0196980

**Published:** 2018-05-10

**Authors:** Silvia De Rosa, Gianluca Villa, Paola Franchi, Aldo Mancino, Federica Tosi, Marina Alessandra Martin, Simona Bazzano, Giorgio Conti, Silvia Maria Pulitanò

**Affiliations:** 1 Department of Anaesthesia and Intensive Care, Catholic University, Rome, Italy; 2 Department of Anaesthesia and Intensive Care, San Bortolo Hospital, Vicenza, Italy; 3 Department of Health Science, Section of Anaesthesiology and Intensive Care, University of Florence, Florence, Italy; 4 Department of Anaesthesiology and Intensive Care, Azienda Ospedaliero Universitaria Careggi, Florence, Italy; University of Bari, ITALY

## Abstract

**Introduction:**

The objective of our present study is to evaluate the impact of different PEEP levels on cerebral hemodynamic, gas exchanges and respiratory system mechanics in paediatric patients with post-traumatic brain swelling treated with decompressive craniectomy (DC).

**Materials and methods:**

A prospective physiologic study was carried out on 14 paediatric patients presenting with severe traumatic brain swelling treated with DC. Transcranial Doppler ultrasonography was performed on the middle cerebral artery bilaterally after DC. After assessment at ZEEP, intracranial pressure (ICP), cerebral perfusion pressure (CPP), mean arterial pressure (MAP), central venous pressure (CVP) and gas exchanges were recorded at PEEP 4 and PEEP 8.

**Results:**

From ZEEP to PEEP 8, the compliance of respiratory system indexed to the weight of the patient significantly increased (P = 0.02) without ICP modifications. No significant variation of the MAP, CPP, Vmed, the total resistance of respiratory system and ohmic resistance of the respiratory system indexed to the weight of the patients was observed. CVP significantly increased between ZEEP and PEEP 8 (P = 0.005), and between PEEP 4 and PEEP 8 (P = 0.05).

**Conclusions:**

PEEP values up to 8 cmH20 seem to be safe in paediatric patients with a severe post-traumatic brain swelling treated with DC.

## Introduction

Traumatic Brain Injury (TBI) is a leading cause of death and disability in children [1,2]. The mechanism of injury in TBI comprises of primary and secondary injuries. The former is the direct consequence of the initial physical insult. Management of severe TBI in critical children secondary brain injury could improve outcome [3]. Although the skull is a rigidly fixed volume compartment, the brain, blood and cerebrospinal fluid (CSF) are relatively incompressible [4]. A steep rise of pressure can affect cerebral blood flow, while secondary insults can arise from systemic factors, hypoxemia and hypotension [5]. The initial mixed metabolic acidosis plus respiratory acidosis and Glasgow Coma Scale Score (GCS) are significant predictors of mortality [6].

Current Brain Trauma recommendations are based on early correction of hypoxemia and avoidance of hypocarbia after severe paediatric TBI [7]. Recruitment manoeuvres with high sustained airway pressures, followed by appropriate positive end-expiratory pressure (PEEP) could be necessary to maintain the recruited alveoli open. High intrathoracic pressure decreases the preload, resulting in diminished cardiac output. Moreover, the impedance of venous blood flow may elevate Intracranial Pressure (ICP) [8]. Increased ICP and brain swelling are the main cause of death in patients with severe TBI. The appropriate treatment of these patients and the balance of benefits and risks represent a complex issue in critical care.

The aim of medical and surgical therapy is to reduce the ICP after TBI: when other treatments have failed, decompressive craniectomy (DC) could allow a rapid decrease of ICP. This procedure allows the relieve intractable intracranial hypertension and/or to prevent or reverse cerebral herniation [9]. The clinical effectiveness of DC in reducing ICP in patients with TBI is under evaluation in current randomized clinical trials, but it has been demonstrated in published clinical investigations [10–13] and in a recent meta-analysis [14]. Although there are a large number of experimental and clinical studies controversy regarding the management of intracranial hypertension and cerebral oedema when a high airway pressure is used [8,15]. there is no evidence regarding the effect of PEEP on cerebral hemodynamic post-DC. Literature involving adult patients suggest to consider decompressive craniectomy, evaluating the PEEP effects on the basis of its influence in reducing the effect of Starling’s resistor significantly [16]. In paediatric populations this effect is uncertain, and the use of PEEP in this situation remains controversial. To the best of our knowledge, no authors have demonstrated the impact of different PEEP levels on cerebral hemodynamic, gas exchanges, and respiratory system mechanics in paediatric patients with a severe post-traumatic brain swelling treated with DC.

## Materials and methods

The present is a single centre observational study. The Institutional Review Board approved the protocol and written informed consent was obtained from parents. Data were collected prospectively on 14 paediatric patients admitted to paediatric intensive care unit (PICU) of Catholic University Medical School, Rome, Italy, between May 2012 and December 2013. Participants were consecutive emergency department patients after diagnosis of severe post-traumatic brain swelling treated by DC in accordance with a standardised protocol. Inclusion criteria were: age between 4 and 16 years, TBI, DC, and the need for postoperative mechanical ventilation. Exclusion criteria were: age younger than 4 years and older than 16 years, cerebral infectious disease, hemodynamic instability requiring inotropic support, and cerebral disease requiring neurosurgery.

### Patient management

During the study period, patients were resuscitated according to institutional practice, which is consistent with the 2012 TBI Guidelines [7,17]. General approach to severe paediatric TBI management includes: sedation and analgesia with midazolam (continuous infusion of 3 to 5 mcg/kg/min) and remifentanil (continuous infusion 0.25 to 0.75 mcg/kg/min); mechanical ventilation was set to provide Volume Control mode (Servo 300; Siemens, Solna, Sweden) and with a fraction of inspired oxygen (FiO_2_) 0.3, a square wave, an inspiratory-expiratory ratio of 1:2, a targeted tidal volume of 7–8 mL/kg and a frequency set to keep the partial pressure of carbon dioxide (PaCO_2_) at 32 to 36 mmHg; intracranial pressure (ICP) monitoring via intraparenchymal catheter or ventriculostomy, care aimed at maintaining ICP at <20 mmHg, cerebral perfusion pressure (CPP) at > 40 mmHg, PaCO_2_ at 35–40 mmHg, SaO_2_ at >90%; core body temperature maintained between 35 and 37.5°C with antipyretics, cooling/warming blankets, or intravascular cooling devices if needed.

The zero hydrostatic references were chosen at the mid-chest for CVP and mean arterial pressure (MAP), and at the external auditory meatus, corresponding to the foramen of Monroe, for ICP and CPP measurements. The head of the bed was kept at 30 degrees for all children. The middle cerebral artery (MCA) mean velocity (Vmed) of the most affected side was determined by Transcranial Doppler ultrasonography (TCD, Multidop X-4, DWL), using a hand-held probe. During the study period, patients with a CPP value below 50 mmHg, and/or an ICP value over 20 mmHg were excluded. When the absence of spontaneous inspiratory efforts was confirmed, the respiratory mechanics were measured at zero end-expiratory pressure (ZEEP). The end-inspiratory occlusion of 3 seconds was obtained by pressing the end-inspiratory hold knob on the ventilator. The ohmic resistance of the respiratory system (RRS _min_), the total resistance of the respiratory system (RRS _max)_, and the compliance of the respiratory system (Crs) were determined. The RRS_min_ is defined as Pmax—P_1_/flow (P_max_ is the peak airway pressure at the end of inspiration; P_1_ is the pressure value at the rapid initial drop after an occlusion manoeuvre). The RRS_max_ is defined as (Pmax—P_2_)/flow (where P_2_ is the pressure value when a plateau is reached after an occlusion manoeuvre longer than 2 s), after subtraction of the value of endotracheal tube resistance. The Crs was calculated as TV expired/P2—PEEP. Respiratory mechanics data were indexed to the weight of the patients expressed in kg. The following parameters were recorded after 20 minutes: MAP, CVP, ICP, CPP, Vmed, and arterial blood gases. After the ZEEP assessment, the increments of PEEP at four and 8 cm H_2_O were applied while the ventilator setting remained unchanged. At each PEEP level, all above-mentioned parameters were recorded (Kleistek ICUlab, Bari, Italy).

### Statistical analysis

Categorical data are presented as percentages, while continuous data as median [25th-75th percentiles]. Considering the pair, not-normal distributed data, the parameters observed at ZEEP for each variable have been compared to those observed at PEEP 4 and PEEP 8 through Friedman test. For those parameters statistically different at Friedman test, a post-hoc analysis with Wilcoxon sign rank test for paired data has been performed between couples of subgroups. A p value of <0.05 was considered statistically significant at Friedman test. Meanwhile p-value adjustment for multiple comparisons (Bonferroni-corrected alphas, 0.05/3 = 0.02) has been considered for post-hoc analysis. Data were analysed using STATA 9.1 software (STATA Corp, 4905, Lakeway Drive College Station, 77845, Texas, US).

## Results

Fourteen patients were enrolled between May 2012 and December 2013. All patients had a TBI with a severe post-traumatic brain swelling treated with DC (S1 Table). During the study, PaCO2 and PaO2/FiO2 ratio remained unchanged over the study period. No patient dropped out at any stage of the study. In the whole population, the application of PEEP (from ZEEP to PEEP 8) significantly increased CrsI (p = 0.02) without ICP modifications (S2 Table). No significant variation was observed for MAP, CPP, Vmed, RRSmaxI, and RRSminI (S1 Fig). CVP significantly increased between ZEEP and PEEP 8 (p = 0.005), and between PEEP 4 and PEEP 8 (p = 0.05) (S2 Table). The median CPP values, calculated as the difference between MAP and CVP, were 69.2 [64.3–73.7]mmHg, 69 [64.9–77.8] mmHg, 67.2 [58.1–72.9] respectively for ZEEP, PEEP 4 and PEEP 8. In patients with reduced CrsI, the application of PEEP did not significantly increased CrsI values up to normal value. In patients with normal CrsI, PEEP-induced a significant increase in CrsI between ZEEP to PEEP 4 and ZEEP to PEEP 8 (P = 0.02)(S3 Table).

## Discussion

The study suggests the impact of different PEEP levels on cerebral hemodynamic, gas exchanges, respiratory system mechanics in paediatric patients with a severe post-traumatic brain swelling treated with DC. PEEP-induced only modest increases in CVP. The application of different PEEP levels, from ZEEP to PEEP 8, induced a modest variation of CrsI associated with modest increases in CVP without ICP variations but also without effect on gas exchanges, respiratory system mechanics, and intracranial hemodynamics.

In a previous studyon children with cerebral neoplasmwe demonstrated that PEEP provides changes in cerebral hemodynamics similar to those described for adults. In particular, PEEP-induced only modest increases in CVP (approximately 1 mm Hg from PEEP 4 to PEEP 8) small ICP rises but also without significant effect on gas exchanges, respiratory system mechanics, and intracranial hemodynamics [18]. a closed skull model, downstream pressure for cerebral perfusion is either ICP or CVP, whichever is the higher. However, with increasing PEEP, we found that CVP calibrated to the level of the heart rises from five to 8 mmHg. In our study, CPP calculated as difference between MAP and ICP resulted 70.3 [60; 72.67], 70.83[67.67; 76.33], and 67.5 [61;74]; respectively for ZEEP, PEEP 4 and PEEP 8. Furthermore, the CPP values, calculated as the difference between MAP and CVP but also MAP and CVP, were 69.2 [64.3–73.7] mmHg, 69 [64.9–77.8] mmHg, 67.2 [58.1–72.9], respectively for ZEEP, PEEP 4 and PEEP 8.

Although, statistical analysis has not been performed, at this level of PEEP the CPP values calculated as CVP-ICP and MAP-CVP are similar.

However, the interactions between the respiratory system and intracranial hemodynamic in brain-injured patients are more complex, and the ventilatory management is challenging. Children with severe TBI could present initial mixed metabolic acidosis plus respiratory acidosis [6]^,^ and it is advocated the early correction of hypoxemia avoiding hypocarbia. In this scenario, the use of PEEP could improve oxygenation, but it could affect intracranial pressure.

Evidence in the literature demonstrates that, with close monitoring of cerebral and systemic haemodynamics, PEEP can be safely applied and titrated to an optimal level in the management of acute respiratory distress syndrome (ARDS) following traumatic brain injury [19]. High PEEP levels increased brain tissue oxygen pressure and oxygen saturation, without an increase in intracranial pressure or decrease in cerebral perfusion pressure. High PEEP levels can be used as a safe alternative to improve brain oxygenation in patients with severe traumatic brain injury and acute respiratory distress syndrome [20]. However, several studies demonstrate that after TBI, cerebral autoregulation is impaired with a severe risk of hypoperfusion. Paediatric patients have small cisterns, cortical sulci are tight and have small ventricles in proportion to the total intracranial volume. The infant's brain has a reduced intracranial compliance [21]. For this reason, children often develop brain swelling defined as a reduction of cerebrospinal spaces, spaces, particularly the basal cisterns on CT scan [16]. DC could be used to treat intracranial pressure preventing or treating herniation [9].

There is no evidence regarding the effect of PEEP on cerebral hemodynamic post-DC.

In patients with normal compliance, PEEP application may raise right atrium pressure increasing internal jugular vein pressure [22]. On the contrary, in patients with low CrsI, this effect is prevented by “stiff lung phenomenon”[16,23]. Infants have high cardiovascular tolerance to the application of high airway pressures. The volume-pressure relationship of the chest wall is much steeper in the infant. For this reason, relatively large changes in intrathoracic pressure cause small variations in chest wall pressure with a limited effect on pleural pressure [24].

In adult patients, authors suggest that any factor likely to reduce the effect of Starling’s resistor significantly, such as decompressive craniectomy, should be considered in the evaluation of PEEP and any effect on the intracranial system [16].

In paediatric patients with decompressive craniectomy, it is uncertain if a small increase in CVP could increase the jugular bulb pressure (Pj) and if a Starling resistor effect acting between dural sinuses and cerebral veins may have dampened it. In addition, when the head is kept at 30 degrees, the vertebral venous plexus may exert cerebral venous drainage better than the jugular veins [18].

This study has several limitations. First, we assumed that the pressure measured at each point might be the same, but it may not be the same in an open skull. Secondly, we did not perform measurements of cardiac output after PEEP applications. Thirdly, we did not perform retrograde cannulation of the internal jugular vein. Thus, we have not prediction of venous drainage impairment. Fourthly, we did not perform the PEEP assessment in children with an increase in ICP or a decrease in CPP.

Fifthly, the sample size was small, and further studies involve large and better-defined population should be performed to confirm our data.

## Conclusion

In conclusion, in the child aged 4–16 years, post-decompressive craniectomy, who has a stable measured ICP (i.e., <20 mm Hg) and stable measured CPP (i.e., >50 mm Hg), the addition of PEEP up to 8 cm H2O does not result in a change in the measurements of ICP or CPP.

Probably, our results could suggest the use of PEEP (up to 8 cm H2O) to restore lung recruitment and improve oxygenation in paediatric patients with severe head injuries.

## Supporting information

S1 Fig CrsI, gas exchanges, MAP, CVP, CPP and Vmed at ZEEP (in white), PEEP4 (in light gray) and PEEP8 (in dark gray). * Statistical significance respect to ZEEP.
† Statistical significance between PEEP4 and PEEP 8.
(JPG)

S1 Table Demographic, clinical, and imaging characteristics of patients with severe brain injury who underwent decompressive craniectomy. BA: Bicycle Accident; GCS: Glascow Coma Score; MVA: Motor Vehicle Accident; PA: Pedestrian Accident; ST: Sport Trauma.
(DOCX)

S2 Table Respiratory mechanics data, MAP, CVP, arterial blood gases, ICP, CPP and Vmed at different PEEP level. * Statistical significance respect to ZEEP.
† Statistical significance between PEEP4 and PEEP 8.
(DOCX)

S3 Table Indexed compliance of respiratory system at different Peep levels in-group with low compliance and normal compliance. * Statistical significance respect to ZEEP.
(DOCX)
