# Supplementary figures and images for: Impact of positive end expiratory pressure on cerebral hemodynamic in paediatric patients with post-traumatic brain swelling treated by surgical decompression
Source: PLoS One. 2018 May 10;13(5):e0196980. doi: 10.1371/journal.pone.0196980 (PMC5944965; doi:10.1371/journal.pone.0196980)

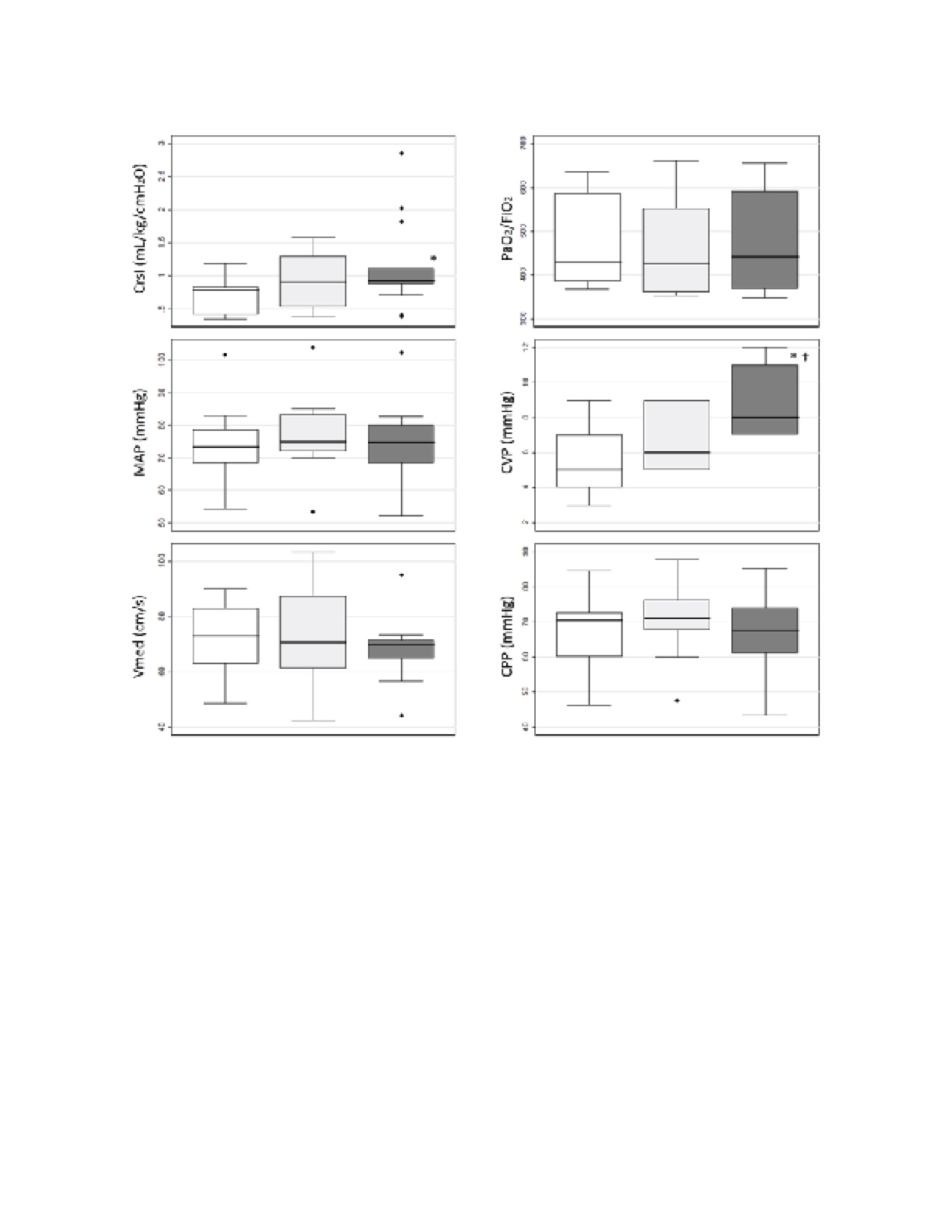

Supplement: S1 Fig — * Statistical significance respect to ZEEP. † Statistical significance between PEEP4 and PEEP 8. (JPG) [file pone.0196980.s001.jpg]
