# Supplementary material for: Impact of positive end expiratory pressure on cerebral hemodynamic in paediatric patients with post-traumatic brain swelling treated by surgical decompression
Source: PLoS One. 2018 May 10;13(5):e0196980. doi: 10.1371/journal.pone.0196980 (PMC5944965; doi:10.1371/journal.pone.0196980)
Supplement: S1 Table — BA: Bicycle Accident; GCS: Glascow Coma Score; MVA: Motor Vehicle Accident; PA: Pedestrian Accident; ST: Sport Trauma. (DOCX) [file pone.0196980.s002.docx]

S1 Table: Demographic, clinical, and imaging characteristics of patients with severe brain injury who underwent decompressive craniectomy*

| Case No. | Age (years) | Weight (kg) | Sex | Cause of Injury | Chest Trauma | Pre-operatory GCS score | Midline Shift on CT scan | Side of Craniectomy |
| --- | --- | --- | --- | --- | --- | --- | --- | --- |
| 1 | 16 | 60 | M | MVA | Yes | 4 | Y | Bilateral |
| 2 | 15 | 60 | M | MVA | No | 6 | Y | Right |
| 3 | 8 | 35 | F | ST | No | 3 | Y | Right |
| 4 | 11 | 40 | F | MVA | No | 3 | Y | Left |
| 5 | 15 | 80 | M | MVA | No | 6 | Y | Left |
| 6 | 4 | 20 | M | PA | No | 3 | Y | Bilateral |
| 7 | 4 | 22 | F | MVA | No | 7 | Y | Bilateral |
| 8 | 14 | 45 | M | MVA | No | 8 | N | Right |
| 9 | 14 | 85 | F | MVA | No | 5 | Y | Left |
| 10 | 7 | 30 | M | MVA | No | 5 | Y | Right |
| 11 | 14 | 50 | M | BA | No | 3 | N | Right |
| 12 | 6 | 20 | F | MVA | Yes | 5 | Y | Left |
| 13 | 11 | 40 | F | BA | No | 6 | Y | Right |
| 14 | 16 | 80 | F | PA | No | 6 | Y | Right |

BA: Bicycle Accident; GCS: Glascow Coma Score; MVA: Motor Vehicle Accident; PA: Pedestrian Accident; ST: Sport Trauma
