# Supplementary material for: Impact of positive end expiratory pressure on cerebral hemodynamic in paediatric patients with post-traumatic brain swelling treated by surgical decompression
Source: PLoS One. 2018 May 10;13(5):e0196980. doi: 10.1371/journal.pone.0196980 (PMC5944965; doi:10.1371/journal.pone.0196980)
Supplement: S2 Table — * Statistical significance respect to ZEEP. † Statistical significance between PEEP4 and PEEP 8. (DOCX) [file pone.0196980.s003.docx]

S2 Table: Respiratory mechanics data, MAP, CVP, arterial blood gases, ICP, CPP and Vmed at different PEEP level.

|  | **ZEEP** | **PEEP 4** | **PEEP 8** | ***Friedman test*** | | ***Post-hoc analysis*** |
| --- | --- | --- | --- | --- | --- | --- |
|  |  |  |  | ***p*** | ***Friedman coeff.*** | ***p*** |
| **CrsI** | 0.79 [0.41; 0.84] | 0.90 [0.53; 1.3] | 0.92 [0.87; 1.12]* | <0.001 | 21.89 | *0.017 |
| **RRSmaxI** | 0.43 [0.13; 1.03] | 0.52 [0.2; 1.2] | 0.57 [0.24 ; 1.45] | 0.10 | 4.54 |  |
| **RRSminI** | 0.29 [0.07; 0.57] | 0.42 [0.1; 1] | 0.45 [0.15; 1.2] | 0.07 | 5.25 |  |
| **PaO2/FiO2** | 427.7 [385; 587.3] | 426 [361; 552.3] | 442 [368.3;592] | 0.40 | 1.86 |  |
| **PaCO2** | 35.3 [32.1; 37.1] | 36.1 [32.9; 37.3] | 132.6 [110.5; 177.6] | 0.07 | 5.29 |  |
| **CPP** | 70.33 [60; 72.67] | 70.83 [67.67; 76.33] | 67.5 [61; 74] | 0.01 | 9.00 |  |
| **ICP** | 6 [5; 9] | 6.5 [5; 10] | 8 [6; 9] | 0.007 | 9.89 |  |
| **MAP** | 73.33 [68.33; 78.67] | 75 [72; 83.33] | 74.66 [68.33; 80] | 0.008 | 9.57 |  |
| **CVP** | 5 [4; 7] | 6 [5;9] | 8 [7; 11] *† | <0.001 | 18.32 | *<0.001  † 0.01 |
| **Vmed** | 72.83 [62.67; 82.67] | 70.5 [61.33; 87.33] | 69.83 [64.67; 71.33] | 0.40 | 1.86 |  |

* Statistical significance respect to ZEEP

† Statistical significance between PEEP4 and PEEP 8
