# Supplementary material for: Impact of positive end expiratory pressure on cerebral hemodynamic in paediatric patients with post-traumatic brain swelling treated by surgical decompression
Source: PLoS One. 2018 May 10;13(5):e0196980. doi: 10.1371/journal.pone.0196980 (PMC5944965; doi:10.1371/journal.pone.0196980)
Supplement: S3 Table — * Statistical significance respect to ZEEP. (DOCX) [file pone.0196980.s004.docx]

**S3 Table**: Indexed compliance of respiratory system at different Peep levels in-group with low compliance and normal compliance.

|  | **ZEEP** | **PEEP4** | **PEEP8** | ***Friedman test*** | | ***Post-hoc analysis*** |
| --- | --- | --- | --- | --- | --- | --- |
|  |  |  |  | ***p*** | ***Friedman coeff.*** | ***p*** |
| CrsI <0.8 | 0.41 [0.37; 0.73] | 0.43 [0.40; 0.53] | 0.87 [0.40; 0.95] | 0.003 | 11.64 |  |
| CrsI>0.8 | 0.84 [0.81; 0.96] | 0.96 [0.90; 1.38]* | 1.07 [0.90; 2.02]* | 0.005 | 10.57 | *0.02 |

* Statistical significance respect to ZEEP
